# Supplementary material for: Complex‐centric proteome profiling by SEC‐SWATH‐MS
Source: Mol Syst Biol. 2019 Jan 14;15(1):e8438. doi: 10.15252/msb.20188438 (PMC6346213; doi:10.15252/msb.20188438)
Supplement: Supplementary file 8 — Dataset EV7 [file MSB-15-e8438-s008.zip › feature_plots_string/Q14691.pdf]

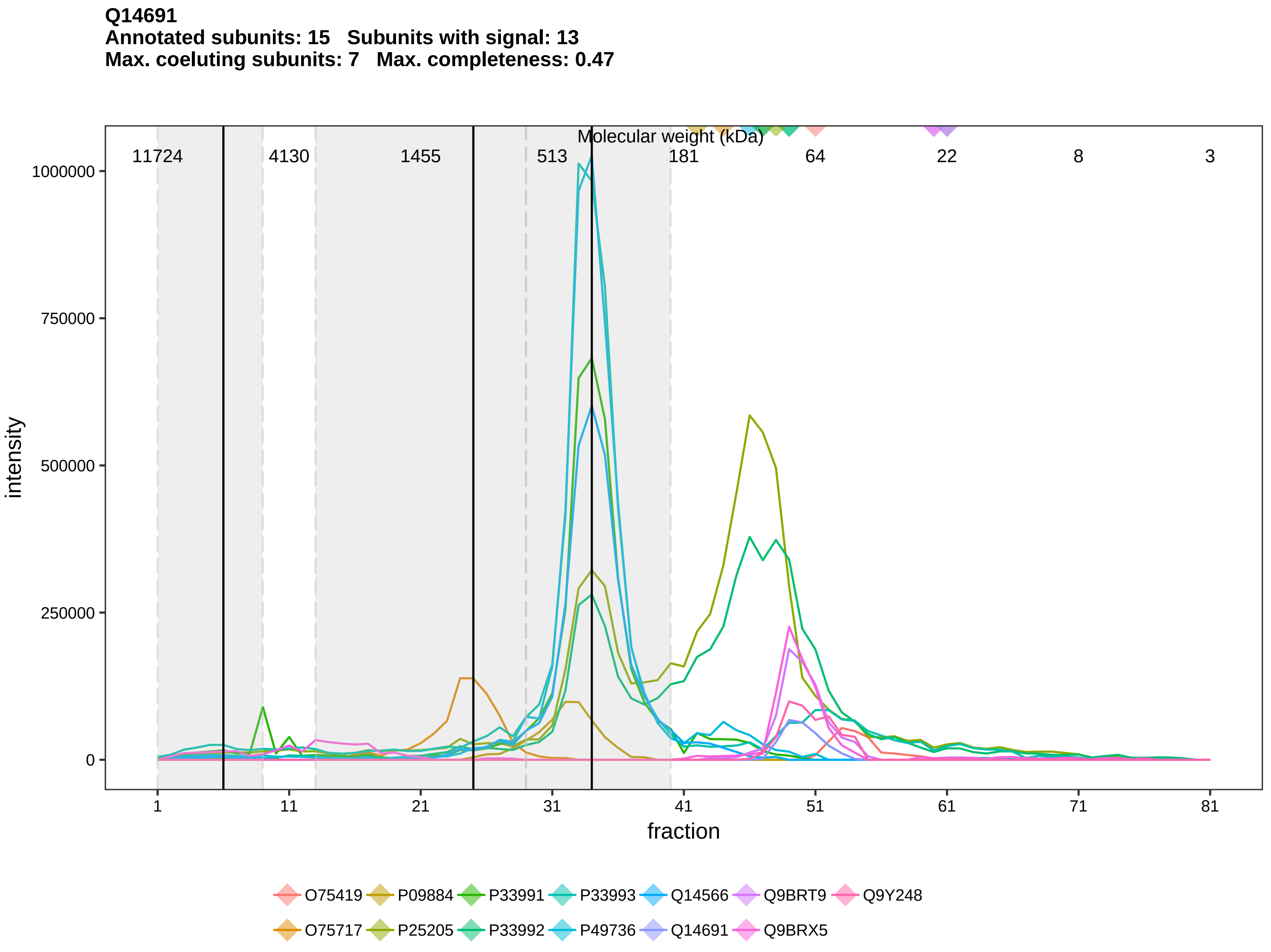
Q14691
Annotated subunits: 15   Subunits with signal: 13
Max. coeluting subunits: 7   Max. completeness: 0.47
Molecular weight (kDa)
11724
4130
1455
513
181
64
22
8
3
1000000
750000
500000
250000
0
intensity
1
11
21
31
41
51
61
71
81
fraction
O75419
P09884
P33991
P33993
Q14566
Q9BRT9
Q9Y248
O75717
P25205
P33992
P49736
Q14691
Q9BRX5
